# Utility of cerebrospinal fluid circulating tumor cell quantification and next-generation sequencing in patients with suspicion for leptomeningeal disease

**DOI:** 10.1093/noajnl/vdag046

**Published:** 2026-03-12

**Authors:** Amanda Onoichenco, Rosivel Galvez, Aaliyah Schultz, Joanna K Tabor, Samuel Latzman, Shoaib A Syed, Naveen Menon, Randy S D’Amico, Morana Vojnic

**Affiliations:** Department of Neurology, Weill Cornell School of Medicine, New York; SUNY Downstate College of Medicine, Brooklyn; SUNY Downstate College of Medicine, Brooklyn; Department of Neurological Surgery, Yale School of Medicine, New Haven; Donald and Barbara Zucker School of Medicine at Hofstra/Northwell, Department of Neurological Surgery, Lenox Hill Hospital, New York; Donald and Barbara Zucker School of Medicine at Hofstra/Northwell, Department of Neurological Surgery, Lenox Hill Hospital, New York; Donald and Barbara Zucker School of Medicine at Hofstra/Northwell, Department of Hematology/Oncology, Lenox Hill Hospital, New York; Donald and Barbara Zucker School of Medicine at Hofstra/Northwell, Department of Neurological Surgery, Lenox Hill Hospital, New York; Rutgers Cancer Institute, New Brunswick

**Keywords:** brain metastasis, circulating tumor cells, leptomeningeal disease, liquid biopsy, next-generation sequencing

## Abstract

**Background:**

Leptomeningeal disease (LMD) is characterized by the spread of cancer to the leptomeninges and cerebrospinal fluid (CSF) and is associated with poor survival. Diagnosing LMD is challenging, as traditional methods such as MRI and CSF cell cytology demonstrate variable sensitivity. This study aims to explore the diagnostic potential of CSF circulating tumor cell (CTC) quantification for LMD in conjunction with current standards.

**Methods:**

This retrospective case series includes 12 patients with suspected LMD who underwent CSF analysis with the CNSide assay for CTC quantification and next-generation sequencing (NGS), alongside MRI and CSF cytology. Relying on a composite definition of LMD-positive, the diagnostic performance of CTC quantification was assessed.

**Results:**

Of 12 patients evaluated for LMD with CNSide, 11 were found to have brain metastases (BM). Lung carcinoma was the most common primary cancer (4/12). Nine patients were deemed LMD-positive based on clinical criteria: 7/9 had a preceding brain metastasis diagnosis, while 2/9 co-presented with LMD and BM. CNSide detected CTCs in 7/12 patients (7/9 of those with LMD) and influenced clinical decision-making by guiding chemotherapy selection and prompting proton craniospinal irradiation. Of the three patients deemed LMD-negative with clinical criteria, all three had negative results on the CNSide assay. The CNSide assay demonstrated a sensitivity of 77.8%, specificity of 100%, and overall accuracy of 83.3%.

**Conclusion:**

The integration of CTC quantification with next-generation sequencing (NGS) can be a valuable adjunct to cell cytology in diagnosing leptomeningeal disease. CSF liquid biopsy may provide earlier detection and inform treatment decisions, ultimately improving patient outcomes.

Key Points

- Leptomeningeal disease is associated with poor survival.
- Current diagnostic standards lack specific, treatment-relevant insights and leave room for ambiguity.
- CSF CTC quantification may facilitate early LMD diagnosis and guide treatment.

Importance of the Study

For many cancer patients, the development of LMD ­represents a devastating and rapid clinical deterioration often with pervasive neurological symptoms. LMD diagnosis poses a clinical challenge as standard technologies lack sensitivity, and the prognosis for LMD is extremely poor with median survival on the scale of weeks. Incorporating CSF CTC quantification alongside MRI and cell cytology has the potential to precipitate earlier diagnosis and intervention with the goal of improving patient outcomes.

LMD is characterized by the infiltration of cancer cells into the leptomeninges and CSF of patients with advanced metastatic disease. This presentation poses a significant challenge in oncology due to the insidious onset and diagnostic complexity. Most cases of LMD originate from non-central nervous system (CNS) solid tumors, particularly lung cancer, melanoma, and, most frequently, breast cancer. LMD affects approximately 5% of cancer patients, though asymptomatic or undiagnosed cases may occur at higher rates.^1–3^ Prognosis remains poor, with median survival ranging from 2 to 4 months after diagnosis, as patients often experience rapid clinical deterioration.^2^

Traditional diagnostic modalities for LMD, including clinical assessment, MRI, and CSF cytology exhibit notable limitations.^4^^,^^5^ While MRI is widely used to identify LMD, other unrelated conditions may result in leptomeningeal enhancement on imaging including radiation, infection, lumbar puncture, and neurosurgical intervention. Given the poor specificity of MRI for LMD, a false positive may harm patients with unnecessary testing and treatment.^6^ CSF cytology, the current diagnostic gold standard, exhibits low-to-moderate sensitivity for LMD, often necessitating multiple lumbar punctures to confirm diagnosis.^5^^,^  ^7^ The limitations of the diagnostic tests described above, as well as their reliance on user-interpretation, reveal a need for improved approaches for detection of LMD.

Recent advances in liquid biopsy techniques have shown promise in improving the early detection of LMD. The quantification of CTCs and circulating tumor DNA (ctDNA) in CSF has been shown to offer higher sensitivity and specificity compared to conventional CSF cytology alone.^8–10^ These biomarkers not only facilitate earlier diagnosis–often preceding MRI or cytology findings–but also provide real-time molecular insights into tumor burden, genetic alterations, and disease progression.^11^ Despite these advancements, challenges remain in optimizing the sensitivity and specificity of liquid biopsy assays, particularly in standardizing methodologies, accounting for variability in CSF sampling, and understanding tumor shedding dynamics in CSF. Further validation in clinical settings is essential to overcome these clinical and technical hurdles.

In this retrospective case series, we present data from 12 patients with suspected brain metastases and LMD, who underwent CSF analysis using the CNSide assay for CTC quantification and NGS. Our goal is to explore the clinical utility of CSF liquid biopsy in LMD diagnosis and its potential to improve patient outcomes by enabling earlier interventions.

## Methods

This retrospective study included 12 consecutive patients presenting to one institution between October 2022 and October 2023 who were suspected of having LMD based on any combination of clinical presentation, suggestive imaging, and presence of brain metastases. CSF samples were obtained and analyzed for cell count, cytology, and CTC quantification with NGS using the CNSide assay. Patient data were collected from electronic medical records, including demographics (age, sex, and ethnicity), primary cancer characteristics (type, molecular profile, date of diagnosis, and stage), brain metastasis status, treatment history, and clinical course. Findings from the Biocept CNSide CSF assay (Biocept, San Diego, CA), including CTC count, molecular markers identified by immunohistochemistry, fluorescence in situ hybridization (FISH), and NGS were also recorded.

### LMD Diagnostic Criteria

Given the low sensitivity of the current gold standard of cell cytology, participants were primarily classified as LMD-positive or negative by a composite definition, requiring either a positive cell cytology or unequivocal findings on MRI (eg leptomeningeal enhancement).^11^^,^^12^ In most cases, diagnosis was complicated by equivocal cytology, therefore, patients were further assessed according to previously established ESMO-EANO LMD criteria as in other clinical studies.^13^^,^^14^ These guidelines stratify LMD-status as “confirmed,” “probable,” “possible,” or “no evidence to suggest” based on the combination of findings on cell cytology, MRI, and clinical symptoms.^5^

### CNSide Assay

The CNSide assay is a liquid biopsy platform developed to detect and characterize circulating tumor cells (CTCs) in a 2.0 mL sample of CSF collected via lumbar puncture or ventriculoperitoneal shunt, as a smaller sample may increase the likelihood of a false negative due to low concentration and lack of CTCs. This method, initially designed for peripheral blood CTC detection, employs the CEE-Sure microfluidic platform, which uses an antibody capture approach.^10^^,^^15^ The assay utilizes a 10-antibody cocktail consisting of anti-EPCAM (anti-epithelial cellular adhesion molecule), anti-TROP2 (anti-trophoblast cell surface antigen 2), anti-FOLR1 (anti-folate receptor alpha), anti-c-MET (anti-tyrosine-protein kinase Met), anti-EGFR (anti-epidermal growth factor receptor), anti-HER2 (anti-human epidermal growth factor receptor 2), anti-MUC1 (anti-mucin 1 cell surface associated), anti-CD318 (anti-CUB domain containing protein 1), anti-SUSD2 (anti-sushi domain containing 2), and anti-CDH2 (anti-cadherin 2). The assay is optimized to minimize cross-reactivity, specifically targeting CTCs in the CSF.^15–17^ In all cases, CNSide testing and conventional CSF cytology were obtained from the same CSF collection event when CNSide was ordered. Repeat cytology at other time points was performed solely at the discretion of the clinical team when a cytology sample resulted in technical processing error or inadequate volume. In patients with repeat CNSide assays, repeat cytology was not uniformly obtained.

### Sample Processing and Analysis

Both fresh and frozen CSF samples were analyzed using NGS and switch blocker analysis. Circulating, cell-free total nucleic acids (cfTNA) were isolated from CSF supernatant using the Qiagen viral total nucleic acid kit (Qiagen, Redwood City, CA, USA). Fresh samples were processed immediately for CTC isolation and molecular analysis.^18^ For CTC identification, the pellet obtained from centrifuged CSF was treated with the 10-antibody cocktail, followed by incubation with biotinylated secondary antibodies.^10^^,^^18^ CNSide employs amplicon-based NGS on cfTNA to identify cancer-related gene mutations (including ALK, BRAF, EGFR, ERBB2, KRAS, MAP2K1, MET, NRAS, PIK3CA, ROS1, and TP53) using Torrent Suite for sequencing and analysis, Ion Reporter for further analysis, and Oncomine Knowledgebase software (ThermoFisher, Waltham, MA, USA) for data annotation and curation.^10^^,^^18^^,^^19^

CNSide identified tumor-associated cells through immunohistochemistry based on the following criteria: positive staining for cytokeratin and 4′,6-diamidino-2-phenylindole (DAPI), as well as the absence of CD45 staining, indicating exclusion of lymphocytes and red blood cells.^10^^,^^18^ Cells positive for CD45 or negative for DAPI were classified as non-tumor cells, including lymphocytes and other CSF components.

### Statistical Analysis

Sensitivity, specificity, positive predictive value, and negative predictive value were calculated to assess the performance of CTC quantification across two cutoffs (CTC/mL > 0 and CTC/mL >1) as validated cutoffs vary depending on cancer type and platform. True positives for LMD were defined by a composite of either positive cell cytology or unequivocal MRI findings.

Sensitivity is calculated as (True Positives)/(True Positives + False Negatives) and represents the percentage of LMD-positive individuals with CTCs detected in the CSF. Specificity is calculated as (True Negatives)/(True Negatives + False Positives) and represents the percentage of LMD-negative individuals without CTCs detected in the CSF. The Positive Predictive Value (PPV) is calculated as (True Positives)/(True Positives + False Positives) and represents the percentage of individuals with CTC detected in the CSF who are LMD-positive. The Negative Predictive Value (NPV) is calculated as (True Negatives)/(True Negatives + False Negatives) and represents the percentage of individuals without CTC detected in the CSF who are LMD-negative. Overall accuracy of the assay was calculated according to (True Positives + True Negatives)/(Total Individuals Tested) and represents the probability that an individual is correctly identified.

## Results

The demographics and clinical features of patients assessed for LMD are presented in Table 1. The cohort included 12 patients (75.0% female, mean age of 60 years). The most common primary cancer was lung carcinoma (4/12, 33.3%), including non-small cell lung adenocarcinoma (*n* = 3) and small cell lung carcinoma (SCLC) (*n* = 1). Other primary cancers included breast cancer (*n* = 3), esophageal carcinoma (*n* = 1), ovarian cancer (*n* = 1), endometrial cancer (*n* = 1), anal squamous cell carcinoma (*n* = 1), and sinonasal neuroendocrine carcinoma (*n* = 1). In all 12 patients, there was significant clinical suspicion for LMD warranting investigation, and 11 of the 12 patients had confirmed parenchymal metastasis either prior to or during LMD investigation.

**Table 1.** Demographics and clinical features of participants

Mean age [Range]		60 [50-78]
**Sex**	Female	9 (75.0%)
	Male	3 (25.0%)
**Race/Ethnicity**	Asian	2 (16.7%)
	Black/African American	1 (8.33%)
	Hispanic	2 (16.7%)
	White	5 (41.7%)
	Other or Unknown	2 (16.7%)
**Primary cancer diagnosis**	Breast	3 (25.0%)
	Lung	4 (33.3%)
	Head/Neck	1 (8.33%)
	Ovarian	1 (8.33%)
	Endometrial	1 (8.33%)
	Anal	1 (8.33%)
	Esophageal	1 (8.33%)
**Brain metastasis (past or present)**	Yes	12 (100%)
**CNSide result for LMD (CTC/mL >0)**	Positive	7 (58.3%)
	Negative	5 (41.7%)
**CNSide result for LMD (CTC/mL >1)**	Positive	4 (33.3%)
	Negative	8 (66.7%)

The clinical course with timeline to development of BM and/or LMD, interventions, and outcomes for each participant are detailed in Table 2. LMD was diagnosed in 9/12 patients: seven had brain metastases that progressed to LMD, two presented with concurrent brain metastases and LMD, and three were LMD-negative. Among the three patients without LMD, all had history of brain metastasis and/or suspicious clinical signs for LMD. Of the six patients who had previously undergone surgical resection for BM, five developed LMD after surgery. Five of the six patients underwent CNSide testing after surgery, with a mean of 239.4 days after resection; the one patient who underwent CNSide prior to surgery had the test 20 days before resection. Among the nine LMD-positive patients for whom outcomes were definitively tracked post-LMD diagnosis, the estimated median survival was 9.3 months.

**Table 2.** Clinical course and outcomes in patients assessed for LMD

Patient (age at biocept collection/sex)	Primary cancer (stage)	Systemic spread	Intracranial disease	Time from primary diagnosis to brain metastasis (months)	Suspicion for LMD	Time from primary diagnosis to LMD (months)	Treatments	Outcome
A (55/F)	Breast (IV)	Lung, liver, adrenal gland, and bilateral lower extremity metastases	Multiple intracranial lesions, diffuse leptomeningeal enhancement	0	Worsening headaches, new nausea and vomiting	8	External beam radiation therapy. Intravenous Methotrexate one cycle, followed by intrathecal methotrexate concurrent with oral tucatinib (HER2 tyrosine kinase inhibitor).	Progression of disease; admitted to hospice 1 year from date of primary diagnosis/4 months after LMD diagnosis.
B (54/F)	Breast (IV)	Bilateral lung metastases	Multiple intracranial lesions, diffuse leptomeningeal enhancement	86	MRI brain with enhancement along periphery of cerebellum	92	Left craniotomy and biopsy of left frontal brain tumors. Transtuzumab deruxtecan, dexamethasone. Referred for proton craniospinal irradiation with failed response.	Patient expired 97 months after primary diagnosis/5 months after LMD diagnosis.
C (59/F)	Non-small cell lung adenocarcinoma (IV)	Spine, L knee metastases	Single intracranial lesion, diffuse leptomeningeal enhancement	9-20^a^	MRI brain with enhancement along surface of R cerebellum	34-45^a^	Lung tumor resection with adjuvant platinum based chemotherapy, after metastatic disease development using erlotinib then resistance led to treatment change to osimertinib. Upon LMD diagnosis capmatinib added to osimertinib.	Suffered a significant decline in her performance status and was lost to follow up. Expired 40-51 months after primary diagnosis and 6 months after LMD diagnosis.
D (78/F)	Serous Ovarian (IV)	Spine metastases	Single intracranial lesion, diffuse leptomeningeal enhancement	72	MRI spine with diffuse leptomeningeal enhancement	83	Total hysterectomy with bilateral salpingo-oophorectomy, and cerebellar metastasis resection. Subsequently niraparib treatment with response until changed to olaparib and bevacizumab. Completed proton craniospinal irradiation,	Patient never recovered her functional capacity and expired 97 months after primary diagnosis/14 months after LMD diagnosis.
E (67/F)	Anal squamous cell (IIIB)	N/A	N/A	0	MRI brain with leptomeningeal enhancement in R cerebellum	N/A-No LMD	Chemotherapy (fluorouracil and mitomycin) and radiation therapy (unspecified) previously maintained on pembrolizumab	Unknown
F (68/F)	Non-small cell lung adenocarcinoma (IV)	N/A	Multiple intracranial lesions	12	Progressive weakness and altered mental status	N/A-No LMD	LLL lobectomy and adjuvant cisplatin/pemetrexed. Whole brain radiation and surgical resection with left temporal craniotomy. Liquid NGS found *EGFR* Exon 18 mutation. Systemic therapy with afatinib, followed by osimertinib. Left frontal craniotomy for resection of tumor with cesium implant.	Unknown
G (54/F)	Breast (IV)	N/A	Single intracranial lesion, diffuse leptomeningeal enhancement	23-34^a^	Gait imbalance and altered mental status	28-39^a^	Right craniotomy for tumor resection, followed by Gamma Knife Radiosurgery (GKRS).	Unknown
H (57/M)	Esophageal adenocarcinoma (IV)	N/A	Multiple intracranial lesions	11	Dizziness, balance issues, falls and diplopia	N/A- No LMD	Previous lumpectomy, chemotherapy (unspecified), and radiosurgery (unspecified) at outside institution, followed by right craniotomy for tumor resection and GKRS at our institution.	Expired 34 months after primary diagnosis/23 months after BM diagnosis.
I (56/M)	Sinonasal neuroendocrine carcinoma (IV)	Diffuse leptomeningeal enhancement (spine)	N/A	12	MRI spine with multiple enhancing lesions in thecal sac	13	Bifrontal craniotomy for resection of anterior skull base mass concurrent with chemotherapy with cisplatin/etoposide. Upon progression underwent stereotactic radiosurgery. Started temozolomide. Received GKRS, proton craniospinal irradiation Capecitabine added to temozolomide Started regorafenib but stopped due to poor tolerance.	Expired 29 months after primary diagnosis/16 months after LMD diagnosis.
J (69/M)	Non-small cell lung adenocarcinoma (IV)	N/A	Single intracranial lesion, diffuse leptomeningeal enhancement	20	MRI brain dural-based occipital calvarium lesion	37	NSCLC treated with middle lobectomy. The patient underwent left suboccipital craniotomy for resection of infratentorial brain metastasis with frameless stereotactic guidance. GKRS, received one dose of ipilimumab/nivolumab, and underwent proton therapy.	Expired 52 months after primary diagnosis/15 months after LMD diagnosis.
K (50/F)	Esophageal adenocarcinoma (IV)	Diffuse leptomeningeal enhancement (spine), hemithorax metastasis (unspecified)	N/A	4	Word-finding difficulty, loss of speech fluency, and encephalopathy	4	Treated with 5FU, oxaliplatin and pembrolizumab, followed by ramucirumab/docetaxel and intrathecal methotrexate.	Patient entered hospice 5 months after date of primary diagnosis/1 month after LMD diagnosis
L (53/F)	Endometrial (IV)	Lung metastasis	Multiple intracranial lesions, diffuse leptomeningeal enhancement	92	MRI brain with enhancement along cerebellar hemispheres, brainstem, sylvian fissures	92	Hysterectomy and chemotherapy (unspecified).VP shunt.	Patient expired 92 months after primary diagnosis/in the same month of BM/LMD diagnosis.

a Discrepancy in months due to unknown month of primary diagnosis.

**Figure 1.**
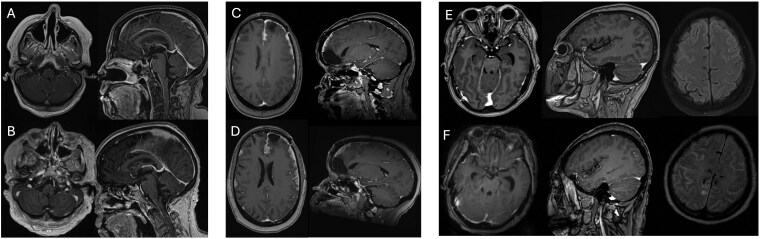
Initial and follow-up brain imaging depicting leptomeningeal disease. (**A)** Initial Brain MRI for Patient A. Findings are consistent with leptomeningeal carcinomatosis. Multiple small foci of metastatic disease mostly in the left frontal lobe with a small lesion also in the left posterior cerebellum adjacent to the dura. No significant mass effect or midline shift. (**B)** 2-month Follow-up Brain MRI for Patient A. Grossly stable supra and infratentorial parenchymal metastases and leptomeningeal carcinomatosis. No new metastases identified. (**C)** Initial Brain MRI for Patient B. Progression of dural disease with thick nodular enhancement increase throughout the bilateral frontal, temporal, occipital regions with increase in edema within the brain. New left frontal, left cerebellar, and basilar cistern leptomeningeal carcinomatosis. Two new enhancing nodules within the right cerebellar hemisphere are likely leptomeningeal in location. (**D)** 2-month Follow-up Brain imaging for Patient B. Since initial imaging, there is increased thickness of numerous nodular dural metastatic deposits throughout the bifrontal, temporal, and occipital regions. There is an increasing edema signal within the subjacent parenchyma as well. **(E)** Initial Brain MRI for Patient C. Few small sites of enhancement are present. One in the left midbrain is 2 mm is concerning for parenchymal metastasis, and few small foci of cerebral enhancement are equivocal for parenchymal or leptomeningeal deposits. Superior cerebellar linear enhancement is somewhat irregular and suspicious for leptomeningeal disease. (**F)** 11-day Follow-up Brain Imaging for Patient C. Interval progression of FLAIR signal abnormality within the subarachnoid spaces with enhancement suspicious for leptomeningeal carcinomatosis. Correlation with CSF sampling is recommended. Stable 2mm nodular focus of enhancement within the midbrain. Slight interval increase in ventricular size within the trans ependymal resorption. Multifocal scattered areas of acute ischemia.

Table 3 details the results for diagnostic evaluation (CSF cytology, MRI imaging, presence of clinical signs), LMD status (according to composite definition and EANO-ESMO classification), and detection of CTCs or NGS variants. Select images representative of LMD on brain imaging may be seen in Figure 1. CTC quantification was performed on all 12 patients with suspicion for LMD regardless of if positive or negative for LMD by composite definition, and the mean CSF volume obtained was 6.18 mL. This allowed for evaluation as a diagnostic tool as well as further exclusion of LMD in patients who were designated “LMD-possible” by EANO-ESMO criteria due to clinical signs, as well as extensive metastatic disease. For patient E, CTC helped confirm negative LMD status despite “LMD-probable” label in the setting of an equivocal MRI.

**Table 3.** Work-up and LMD status in high-risk patients

Patient (age at biocept collection/sex)	Primary cancer (stage)	Method and volume (mL) of CSF collection	Cell cytology	Suspicion of LMD on MRI imaging	Clinical signs present?	LMD-status per composite definition	**LMD-status per EANO–ESMO classification** ^a^	CNSide for CTCs	Next-generation sequencing
A (55/F)	Breast (IV)	LP; 2.70	Equivocal	Yes (Figure 1A and B)	Yes	Positive	II—probable	Detected	Negative
B (54/F)	Breast (IV)	LP; 5.90	Negative	Yes—nodular (Figure 1C and D)	Yes	Positive	IIB—probable	Detected	NGS on CSF not completed
C (59/F)	Non-small cell lung adenocarcinoma (IV)	LP; 7.60	Equivocal	Yes (Figure 1E and 1F)	Yes	Positive	II—probable	Detected	Variants detected
D (78/F)	Serous Ovarian (IV)	LP; 7.30	Equivocal	Yes	Yes	Positive	II—probable	Detected	Variants detected
E (67/F)	Anal squamous cell (IIIB)	LP; 7.80	Negative	Yes- equivocal	Yes	Negative	II—probable	Negative	Negative
F (68/F)	Non-small cell lung adenocarcinoma (IV)	LP; 6.80	Negative	No	Yes	Negative	IID- possible	Negative	Negative
G (54/F)	Breast (IV)	LP; 7.80	Negative	Yes	Yes	Positive	II—probable	Negative	Variants detected
H (57/M)	Esophageal adenocarcinoma (IV)	VPS; 1.60	Negative	No	Yes	Negative	IID- possible	Negative	Negative
I (56/M)	Sinonasal neuroendocrine carcinoma (IV)	LP; 7.20	Equivocal	Yes	Yes	Positive	II—probable	Negative	Negative
J (69/M)	Non-small cell lung adenocarcinoma (IV)	VPS; 6.50	Negative	Yes	No	Positive	II—possible	Detected, then negative on repeat	Negative
K (50/F)	Esophageal adenocarcinoma (IV)	LP; 5.90	Equivocal	Yes	Yes	Positive	II—probable	Detected	NGS on CSF not completed
L (53/F)	Endometrial (IV)	VPS; 7.00	Negative	Yes	Yes	Positive	II—probable	Detected	Variants detected

Abbreviations: LP, Lumbar Puncture; VPS, Ventriculoperitoneal Shunt.

a EANO–ESMO Classification limited due to lack of reported MRI descriptors including linear versus nodular LMD.

Table 4 lists detailed results for patients with positive CTC reports or NGS results (*n* = 8) and excludes negative testing results. Positive CTC results and/or detection of NGS variants influenced treatment decisions guiding chemotherapy selection and radiation therapy referrals (Table 2). For example, in patient A, serial testing demonstrated a decrease in CTCs, indicating a positive response to intrathecal chemotherapy. In patients B, D, and J, CTCs reaffirmed LMD in patients with MRI findings suggestive of the disease, leading to referrals for proton craniospinal irradiation. In patient B, CTC testing identified LMD before it was detected by MRI, allowing for earlier therapeutic intervention. Patient C showed an increasing trend in CTCs, which suggested a lack of treatment response; NGS allowed for the discovery of *MET* amplification and a resistance to EGFR TKI prompting a change in therapeutic strategy to a combination therapy of capmatinib and osimertinib.

**Table 4.** Biocept results for patients with positive CTC reports or NGS results; patients with negative CTC and NGS not listed

Patient	Primary cancer	Molecular profile of primary	Biocept collection day	Total tumor Cells	Tumor cells per mL	CK+ tumor cells	ICC detected	ICC not detected	FISH detected	FISH not detected	NGS variants
A	Breast	Triple negative	Initial- day 0	2	1.1	2	None	PR	None	HER2, NTRK1, NTRK3	No
			Repeat- day 6	31	3.97	30		ER, PR, PD-L1	HER2	FGFR1, NTRK1, NTRK3	
			Repeat- day 14	14	1.79	10		ER, PR, PD-L1	None	HER2, NTRK1, NTRK3, FGFR1	
			Repeat- day 23	23	3.11	23		ER, PR, PD-L1	HER2	FGFR1, NTRK1, NTRK3	
			Repeat—day 35	19	2.57	19		ER, PR, PD-L1	None	HER2, NTRK1, NTRK3, FGFR1	
B	Breast	ER/PR negative, HER2 low	Initial- day 0	5	0.85	4	None	ER, PD-L1	Inconclusive	Inconclusive	N/A
C	Lung	EGFR exon 19 del, C797S lung adenocarcinoma	Initial- day 0	826	119.71	826	None	PD-L1	ALK, CMET	NTRK3, ROS1, RET, NTRK1	*EGFR* exon 19 del, *TP53* H214R
			Repeat- day 35	3410	448.68	3369		PD-L1	CMET	NTRK3, ALK, ROS1, RET, NTRK1	*EGFR* exon 19 del, *TP53* H214R
			Repeat- day 57	4112	527.18	4100		PD-L1	CMET	NTRK3, ALK, ROS1, RET, NTRK1	*EGFR* exon 19 del, *TP53* H214R
D	Ovarian	BRCA1 mutant, HRD positive	Initial- day 0	3315	454.11	3241	None	PD-L1	HER2	N/A	BRCA1 G1706, TP53 A161T, MTOR, G1384S, TRAF7 K321N, CDKN1B Amplification, RECQL Amplification, CCND2 Amplification, DDR2 Amplification, NTRK1 Amplification, RIT1 Amplification, SDHC Amplification, PMS2 Deletion, RAC1 Deletion, RASA1 Deletion, CCND2 Amplification, DDR2 Amplification, NTRK1 Amplification, RIT1 Amplification, SDHC Amplification, PMS2 Deletion, RAC1 Deletion, RASA1 Deletion, CCNE L346R, KRAS Amplification, CD276 V330M, CSDE1 L346R, KRAS Amplification, CD276 V330M, CSDE1 K457I, DROSHA 7247M, FOXA1 E255, KMT2C K4887_F4888del, ETV6 Amplification, PIK3C2G Amplification, MED12 Deletion, ATRX Deletion, CDH1 Deletion, FTSJD1 Deletion, ZFHX3 Deletion, FLCG2 Deletion, FOXF1 Deletion, ANKRD11 Deletion, FANCA Deletion, MAP3K1 Deletion, PLK2 Deletion, CARD11 Deletion, ETV1 Deletion, NKX3-1 Deletion, DUSP4 Deletion, NSD3 Deletion, FGFR1 Deletion, BTK Deletion, PIK3C Deletion, SMAD2 Deletion, SMAD4 Deletion, MALT1 Deletion, PMAIP1 Deletion, BCL2 Deletion, SERPINB4 Deletion, SERPINB3 Deletion, SOS1 Deletion, ABRAXAS1 Deletion, EIF4E Deletion, TET2 Deletion
G	Breast		Initial- day 0	0	0	0	N/A	N/A	N/A	N/A	*HER2* amplification, *TP53* R213L
J	Lung	- KEAP1 G509W, STK11 exon 1 loss, APC G721*, PD-L1 negative	Initial- day 0	4	0.62	3	None	PD-L1	None	ALK, POS1, CMET, RET, NTRK1, NTRK3	None
			Repeat- day 48	0	0	0	N/A	N/A	N/A	N/A	None
			Repeat- day 56	0	0	0	N/A	N/A	N/A	N/A	None
K	Esophageal adenocarcinoma		Initial- day 0	84 092	12 252.88	84 092	none	ER, PD-L1, PR	HER2	NTRK1, NTRK3	NGS on CSF not completed
L	Endometrial		Initial- day 0	4	0.57	2	none	ER, PD-L1, PR	none	HER2, NTRK1, NTRK3, FGFR1	NGS on CSF not completed

Using a cut off of CTC/mL > 0, the CNSide assay demonstrated a sensitivity of 77.8%, specificity of 100%, PPV of 100%, NPV of 60.0%, and an overall accuracy of 83.3%. Overall, CTCs were detected in 7/12 patients, all of whom were confirmed as LMD-positive by composite definition requiring either positive CSF cytology or unequivocal MRI findings. Among the five patients who tested negative for CTCs, CNSide failed to identify CTCs in two LMD-positive patients. This was recalculated using a cutoff of CTC/mL >1 to show a sensitivity of 44.4%, specificity of 100%, PPV of 100%, NPV of 37.5%, and an overall accuracy of 58.3%; through raising the cutoff, CNSide failed to identify five LMD-positive patients.

## Discussion

Leptomeningeal disease represents a devastating complication of cancer progression, characterized by the spread of malignant cells to the leptomeningeal layers. LMD occurs in approximately 5% of cancer patients, though this number may be underreported due to asymptomatic or undiagnosed cases.^1^ While LMD can develop either concurrently with or independently of brain metastases, the risk of LMD is significantly increased following surgical resection.^2^ In our case series, five of six patients with a history of brain tumor resection developed LMD, supporting previous literature that identifies BM resection as a risk factor for LMD and further informing the target demographic for LMD evaluation. The prognosis for LMD is extremely poor with a reported median survival of 4-6 weeks without treatment. With existing interventions like intrathecal chemotherapy and radiation therapy, a patient’s life may be prolonged by only months with literature reporting an extended median survival of 3-6 months.^20^ In contrast, the patients in the present case series experienced slightly longer median survival of approximately 9.3 months^2^ (Table 2). Overall, the advent of circulating tumor cell quantification technology and next-generation sequencing is a promising supplement to existing diagnostic methods, making possible earlier intervention and more personalized chemotherapy regimens.

CSF cytology, the current gold standard for LMD diagnosis, is known for its high specificity but low-to-moderate sensitivity. The sensitivity of CSF cytology ranges from 50% to 75% at first puncture, improving to 80%-90% with repeat or high-volume sampling.^10^ Despite its utility, CSF cytology is limited by its dependence on the collection technique and sample quality.^7^ Our findings corroborate these limitations, as none of the CSF cytology definitively confirmed LMD; findings were at most equivocal for LMD-positive cases (*n* = 5) and failed to detect four LMD-positive patients, highlighting the need for more reliable, less invasive diagnostic methods for LMD. MRI is an important diagnostic tool for LMD, revealing signs such as leptomeningeal enhancement or subarachnoid nodules. However, MRI has inconsistent sensitivity across different studies with reported ranges of 76%-100% and the diagnostic criteria for LMD vary in clinical practice.^7^^,^^12^ Neurological symptoms, including cranial nerve palsies, radiculopathies, and gait disturbances can be suggestive of LMD; however, their clinical presentation is often varied. Current diagnostic standards for LMD acknowledge the role of a multi-modal approach; per the EANO-ESMO classification system, when gold standard cell cytology is negative or equivocal, probable LMD diagnosis may be evoked with the combination of MRI findings and consistent clinical symptoms.^5^

In response to the limitations of MRI and CSF cytology, liquid biopsy, specifically the detection of CTCs and ctDNA in CSF, is a promising adjunct to traditional methods. Liquid biopsy offers several advantages over traditional methods, including higher sensitivity and the ability to provide molecular insights to guide treatment decisions. In our study, the Biocept CNSide assay demonstrated diagnostic utility comparable to MRI and CSF cytology, while also providing molecular insights that directly impacted treatment decisions. In this cohort, the sensitivity was 77.8% and specificity 100%, in line with published data suggesting that CTC-based liquid biopsy can have a sensitivity of up to 93%-94%, higher than CSF cytology, and specificity as high as 95%-100%.^11^^,^^14^ Specifically, CNSide detected CTCs in patients K and L that ultimately led to correct, positive LMD diagnoses despite originally negative cell cytology findings. Thus, CNSide can confer additional levels of confirmation and confidence in LMD diagnosis, identifying possible false negatives and serving as more of an adjunctive, complementary check on diagnostic cell cytology than a replacement.

In addition to its diagnostic utility, CTC quantification offers a valuable tool for monitoring disease burden and treatment response. Further research is needed for the role of CTC-quantification in monitoring treatment response and disease progression; standards currently proposed by the Response Assessment in Neuro-Oncology (RANO) group, suggest progression in either clinical presentation, craniospinal MRI, or CSF cytology is adequate, regardless of stable findings across other modalities.^4^^,^^5^ In our series, CTC levels tracked patient outcomes in real time, as evidenced by patient A for whom a decrease in CTCs was indicative of a positive response to chemotherapy, and patient C for whom increasing CTC levels suggested a lack of therapeutic efficacy. This ability to monitor disease progression through serial CTC measurements has been demonstrated in prior studies, where increased CTC levels predicted disease progression months before MRI or CSF cytology results became positive.^8^ Furthermore, the use of NGS on CTCs allows for the identification of actionable mutations: in patient C, the detection of an EGFR deletion and MET amplification prompted initiation of oral capmatinib (MET TKI) and osimertinib. This demonstrates the utility of liquid biopsy not only in diagnosing LMD but also in providing personalized treatment options.

It is important to note that genomic alterations detected by ctDNA NGS may diverge from those identified on primary tumor tissue NGS because of tumor heterogeneity, treatment driven clonal selection, and compartment-specific tumor shedding, with important implications for treatment selection. Brastianos et al identified that brain metastases and their paired primary systemic tumors share a common ancestor but then evolve along separate branches, producing metastasis-specific genomic changes.^3^ This translates into potentially actionable alterations enriched in brain metastases that were not always captured in the primary tumor, highlighting the risk of relying on a single tissue specimen for therapeutic decisions. Therefore, discordance between alterations detected by ctDNA NGS and primary tumor tissue NGS is a clinically meaningful possibility that should be explicitly considered when interpreting genomic results and selecting therapy.^21^^,^^22^ Integrating liquid biopsy results with tissue from the site of active disease can mitigate this concern by refining targeted therapy selection based on both intracranial and systemic samples.

Unlike MRI and CSF cytology, CTC analysis serves as a prognostic and therapeutic response marker. In a clinical trial using the CellSearch system, prospective CTC analysis in HER2+ breast cancer patients with LMD accurately predicted disease burden and progression during intrathecal trastuzumab treatment. Notably, three out of fourteen patients in the trial exhibited increased CSF CTC levels 2-3 months before MRI-detected changes, while CSF cytology remained negative.^8^ This underscores the potential of CSF liquid biopsy to assess LMD disease burden early, enabling timely treatment adjustments. Furthermore, CSF-CTC quantification via the CellSearch platform was shown to better predict survival outcomes compared to CSF cytology and MRI findings in patients newly diagnosed with LMD.^9^ In addition to CTC analysis, quantification of tumor markers (TM) in the CSF, such as CYFRA 21-1, CEA, CA125, CA19-9, and CA153, has proven to be useful. Notably, CYFRA 21-1 may exhibit diagnostic concentrations in the CSF even when serum levels remain normal.^23^

Despite the promising results of our study, there are several limitations. The assay was used at our center for the one-year period during which it was available to our service. The relatively small sample size and retrospective design–constrained by the patient population size presenting with LMD suspicion to our institution–limit the generalizability of our findings. Larger, prospective studies are necessary to confirm the diagnostic and prognostic value of CTC-based assays in LMD. While some studies evaluate CTC quantification in direct comparison to the gold standard, our cell cytology evaluation yielded only negative or equivocal results. This lack of true positives limited our evaluation and highlighted the need for a composite definition of LMD incorporating MRI findings. Moreover, the CNSide assay has a published specificity of 95%, yielding a false positive rate of 5%.^17^ Thus, false positives may result from assay failures or sample contamination from existing brain metastases physically located near CSF drainage pathways in patients that do not yet meet the criteria for LMD.^24^ Additionally, the CNSide assay returned negative NGS results–no targetable mutations–in three LMD-positive patients (Patients A, I, and J), representing a technical, treatment-related limitation of the assay. Further, our composite definition of LMD-positive did not allow for independent evaluation of cell cytology and MRI sensitivity/specificity, and in cases of repeat CNSide assays, repeat cytology was not uniformly obtained.

While the Biocept CNSide assay demonstrated clinical utility, its sensitivity in this patient cohort was lower than that reported for MRI or cell cytology. The reported sensitivities of 77.8% and 44.4% for CTC/mL > 0 and CTC/mL > 1, respectively, are much lower than the accepted CNSide sensitivity of 92%.^17^ Factors such as tumor heterogeneity, small sample size, and variability in CTC shedding may have contributed to the observed false negatives and the resulting, outlier-level low sensitivity. Calculations were performed across two cutoffs as validated cutoff values vary depending on cancer type; for instance, CTC/mL > 0.9 CTC/mL is verified for NSCLC while a CTC/mL > 1 may be used for breast, lung, mixed solid tumors, and her2+ solid tumors.^8^^,^^11^^,^^12^^,^^25^ Establishing standardized thresholds for CTC detection and clear clinical guidelines for their use will be essential for widespread adoption of this technology.

Our findings support the role of CTC-based liquid biopsy in LMD diagnosis and management. By enabling earlier detection and guiding molecularly targeted therapies, liquid biopsy represents a valuable clinical tool alongside MRI and CSF cytology. Further studies are warranted to refine its diagnostic accuracy and establish clinical guidelines for routine implementation.

## Supplementary Material

vdag046_Supplementary_Data

## Data Availability

Data will be made available upon request.
