# Supplementary material for: Utility of cerebrospinal fluid circulating tumor cell quantification and next-generation sequencing in patients with suspicion for leptomeningeal disease
Source: Neurooncol Adv. 2026 Mar 12;8(1):vdag046. doi: 10.1093/noajnl/vdag046 (PMC13036599; doi:10.1093/noajnl/vdag046)
Supplement: vdag046_Supplementary_Data [file vdag046_supplementary_data.docx]

| **Mean age [Range]** |  | 60 [50-78] |  |
| --- | --- | --- | --- |
|  |  |  |  |
| **Sex** | Female | 9 (75.0%) |  |
|  | Male | 3 (25.0%) |  |
| **Race/Ethnicity** | Asian | 2 (16.7%) |  |
|  | Black/ African American | 1 (8.33%) |  |
|  | Hispanic | 2 (16.7%) |  |
|  | White | 5 (41.7%) |  |
|  | Other or Unknown | 2 (16.7%) |  |
| **Primary Cancer Diagnosis** | Breast | 3 (25.0%) |  |
|  | Lung | 4 (33.3%) |  |
|  | Head/Neck | 1 (8.33%) |  |
|  | Ovarian | 1 (8.33%) | |
|  | Endometrial | 1 (8.33%) | |
|  | Anal | 1 (8.33%) |  |
|  | Esophageal | 1 (8.33%) |  |
| **Brain Metastasis (past or present)** | Yes | 12 (100%) |  |
| **CNSide™ Result for LMD (CTC/mL >0)** | Positive | 7 (58.3%) |  |
|  | Negative | 5 (41.7%) |  |
| **CNSide™ Result for LMD (CTC/mL >1)** | Positive | 4 (33.3%) |  |
|  | Negative | 8 (66.7%) |  |

Table 1: Demographics and clinical features of participants

| **Patient (Age at Biocept Collection/ Sex))** | **Primary Cancer (stage)** | **Systemic Spread** | **Intracranial Disease** | **Time from Primary Diagnosis to Brain Metastasis (months)** | **Suspicion for LMD** | **Time from Primary Diagnosis to LMD (months)** | **Treatments** | **Outcome** |
| --- | --- | --- | --- | --- | --- | --- | --- | --- |
| A  (55/F) | Breast (IV) | Lung, liver, adrenal gland, and bilateral lower extremity metastases | Multiple intracranial lesions, diffuse leptomeningeal enhancement | 0 | Worsening headaches, new nausea and vomiting | 8 | External beam radiation therapy. Intravenous Methotrexate one cycle, followed by intrathecal methotrexate concurrent with oral tucatinib (HER2 tyrosine kinase inhibitor). | Progression of disease; admitted to hospice 1 year from date of primary diagnosis/ 4 months after LMD diagnosis. |
| B  (54/F) | Breast (IV) | Bilateral lung metastases | Multiple intracranial lesions, diffuse leptomeningeal enhancement | 86 | MRI brain with enhancement along periphery of cerebellum | 92 | Left craniotomy and biopsy of left frontal brain tumors.  Transtuzumab deruxtecan, dexamethasone. Referred for proton craniospinal irradiation with failed response. | Patient expired 97 months after primary diagnosis/ 5 months after LMD diagnosis. |
| C  (59/F) | Non-small cell lung adenocarcinoma (IV) | Spine, L knee metastases | Single intracranial lesion, diffuse leptomeningeal enhancement | 9-20^a^ | MRI brain with enhancement along surface of R cerebellum | 34-45^a^ | Lung tumor resection with adjuvant platinum based chemotherapy, after metastatic disease development using erlotinib then resistance led to treatment change to osimertinib. Upon LMD diagnosis capmatinib added to osimertinib. | Suffered a significant decline in her performance status and was lost to follow up. Expired 40-51 months after primary diagnosis and 6 months after LMD diagnosis. |
| D  (78/F) | Serous Ovarian (IV) | Spine metastases | Single intracranial lesion, diffuse leptomeningeal enhancement | 72 | MRI spine with  diffuse leptomeningeal enhancement | 83 | Total hysterectomy with bilateral salpingo-oophorectomy, and cerebellar metastasis resection.  Subsequently niraparib treatment with response until changed to olaparib and bevacizumab. Completed proton craniospinal irradiation, | Patient never recovered her functional capacity and expired 97 months after primary diagnosis / 14 months after LMD diagnosis. |
| E  (67/F) | Anal squamous cell (IIIB) | N/A | N/A | 0 | MRI brain with leptomeningeal enhancement in R cerebellum | N/A-No LMD | Chemotherapy (fluorouracil and mitomycin) and radiation therapy (unspecified) previously maintained on pembrolizumab | Unknown |
| F  (68/F) | Non-small cell lung adenocarcinoma (IV) | N/A | Multiple intracranial lesions | 12 | Progressive weakness and altered mental status | N/A-No LMD | LLL lobectomy and adjuvant cisplatin/pemetrexed. Whole brain radiation and surgical resection with left temporal craniotomy.  Liquid NGS found *EGFR* Exon 18 mutation. Systemic therapy with afatinib, followed by osimertinib.  Left frontal craniotomy for resection of tumor with cesium implant. | Unknown |
| G  (54/F) | Breast (IV) | N/A | Single intracranial lesion, diffuse leptomeningeal enhancement | 23-34^a^ | Gait imbalance and altered mental status | 28-39^a^ | Right craniotomy for tumor resection, followed by Gamma Knife Radiosurgery (GKRS). | Unknown |
| H  (57/M) | Esophageal adenocarcinoma (IV) | N/A | Multiple intracranial lesions | 11 | Dizziness, balance issues, falls and diplopia | N/A- No LMD | Previous lumpectomy, chemotherapy (unspecified), and radiosurgery (unspecified) at outside institution, followed by right craniotomy for tumor resection and GKRS at our institution. | Expired 34 months after primary diagnosis/ 23 months after BM diagnosis. |
| I  (56/M) | Sinonasal neuroendocrine carcinoma (IV) | Diffuse leptomeningeal enhancement (spine) | N/A | 12 | MRI spine with multiple enhancing lesions in thecal sac | 13 | Bifrontal craniotomy for resection of anterior skull base mass concurrent with chemotherapy with cisplatin/etoposide.  Upon progression underwent stereotactic radiosurgery. Started temozolomide.  Received GKRS, proton craniospinal irradiation  capecitabine added to temozolomide  Started regorafenib but stopped due to poor tolerance. | Expired 29 months after primary diagnosis/ 16 months after LMD diagnosis. |
| J  (69/M) | Non-small cell lung adenocarcinoma (IV) | N/A | Single intracranial lesion, diffuse leptomeningeal enhancement | 20 | MRI brain dural-based occipital calvarium lesion | 37 | NSCLC treated with middle lobectomy. The patient underwent left suboccipital craniotomy for resection of infratentorial brain metastasis with frameless stereotactic guidance.  GKRS, received one dose of ipilimumab/nivolumab, and underwent proton therapy. | Expired 52 months after primary diagnosis/ 15 months after LMD diagnosis. |
| K  (50/F) | Esophageal adenocarcinoma (IV) | Diffuse leptomeningeal enhancement (spine), hemithorax metastasis (unspecified) | N/A | 4 | Word-finding difficulty, loss of speech fluency, and encephalopathy | 4 | Treated with 5FU, oxaliplatin and pembrolizumab, followed by ramucirumab/docetaxel and intrathecal methotrexate. | Patient entered hospice 5 months after date of primary diagnosis/ 1 month after LMD diagnosis |
| L  (53/F) | Endometrial (IV) | Lung metastasis | Multiple intracranial lesions, diffuse leptomeningeal enhancement | 92 | MRI brain with enhancement along cerebellar hemispheres, brainstem, sylvian fissures | 92 | Hysterectomy and chemotherapy (unspecified).  VP shunt. | Patient expired 92 months after primary diagnosis/ in the same month of BM/LMD diagnosis. |

Table 2: Clinical course and outcomes in patients assessed for LMD.

^a^ Discrepancy in months due to unknown month of primary diagnosis

| **Patient (Age at Biocept Collection/ Sex))** | **Primary Cancer (stage)** | **Method and Volume (mL) of CSF Collection** | **Cell Cytology** | **Suspicion of LMD on MRI Imaging** | **Clinical Signs Present?** | **LMD-status per Composite Definition** | **LMD-status per EANO–ESMO Classification ^a^** | **CNSide for CTCs** | **Next-Generation Sequencing** |
| --- | --- | --- | --- | --- | --- | --- | --- | --- | --- |
| A  (55/F) | Breast (IV) | LP; 2.70 | Equivocal | Yes (Figure 1a, Figure 1b) | Yes | Positive | II - probable | Detected | Negative |
| B  (54/F) | Breast (IV) | LP; 5.90 | Negative | Yes - nodular (Figure 2a, Figure 2b) | Yes | Positive | IIB - probable | Detected | NGS on CSF not completed |
| C  (59/F) | Non-small cell lung adenocarcinoma (IV) | LP; 7.60 | Equivocal | Yes (Figure 3a, Figure 3b) | Yes | Positive | II - probable | Detected | Variants detected |
| D  (78/F) | Serous Ovarian (IV) | LP; 7.30 | Equivocal | Yes | Yes | Positive | II - probable | Detected | Variants detected |
| E  (67/F) | Anal squamous cell (IIIB) | LP; 7.80 | Negative | Yes- equivocal | Yes | Negative | II - probable | Negative | Negative |
| F  (68/F) | Non-small cell lung adenocarcinoma (IV) | LP; 6.80 | Negative | No | Yes | Negative | IID- possible | Negative | Negative |
| G  (54/F) | Breast (IV) | LP; 7.80 | Negative | Yes | Yes | Positive | II - probable | Negative | Variants detected |
| H  (57/M) | Esophageal adenocarcinoma (IV) | VPS; 1.60 | Negative | No | Yes | Negative | IID- possible | Negative | Negative |
| I  (56/M) | Sinonasal neuroendocrine carcinoma (IV) | LP; 7.20 | Equivocal | Yes | Yes | Positive | II - probable | Negative | Negative |
| J  (69/M) | Non-small cell lung adenocarcinoma (IV) | VPS; 6.50 | Negative | Yes | No | Positive | II - possible | Detected, then negative on repeat | Negative |
| K  (50/F) | Esophageal adenocarcinoma (IV) | LP; 5.90 | Equivocal | Yes | Yes | Positive | II - probable | Detected | NGS on CSF not completed |
| L  (53/F) | Endometrial (IV) | VPS; 7.00 | Negative | Yes | Yes | Positive | II - probable | Detected | Variants detected |

Table 3:  Work-up and LMD status in high-risk patients

^a^EANO–ESMO Classification limited due to lack of reported MRI descriptors including linear vs nodular LMD.

LP: Lumbar Puncture. VPS: Ventriculoperitoneal Shunt.

| **Patient** | **Primary Cancer** | **Molecular Profile of Primary** | **Biocept Collection Day** | **Total Tumor Cells** | **Tumor Cells per mL** | **CK+ Tumor Cells** | **ICC detected** | **ICC not detected** | **FISH detected** | **FISH not detected** | **NGS Variants** |
| --- | --- | --- | --- | --- | --- | --- | --- | --- | --- | --- | --- |
| A | Breast | Triple negative | Initial- day 0 | 2 | 1.1 | 2 | None | PR | None | HER2, NTRK1, NTRK3 | No |
|  |  |  | Repeat- day 6 | 31 | 3.97 | 30 |  | ER, PR, PD-L1 | HER2 | FGFR1, NTRK1, NTRK3 |  |
|  |  |  | Repeat- day 14 | 14 | 1.79 | 10 |  | ER, PR, PD-L1 | None | HER2, NTRK1, NTRK3, FGFR1 |  |
|  |  |  | Repeat- day 23 | 23 | 3.11 | 23 |  | ER, PR, PD-L1 | HER2 | FGFR1, NTRK1, NTRK3 |  |
|  |  |  | Repeat – day 35 | 19 | 2.57 | 19 |  | ER, PR, PD-L1 | None | HER2, NTRK1, NTRK3, FGFR1 |  |
| B | Breast | ER/PR negative, HER2 low | Initial- day 0 | 5 | 0.85 | 4 | None | ER, PD-L1 | Inconclusive | Inconclusive | N/A |
| C | Lung | EGFR exon 19 del, C797S lung adenocarcinoma | Initial- day 0 | 826 | 119.71 | 826 | None | PD-L1 | ALK, CMET^b^ | NTRK3, ROS1, RET, NTRK1 | *EGFR* exon 19 del, *TP53* H214R |
|  |  |  | Repeat- day 35 | 3410 | 448.68 | 3369 |  | PD-L1 | CMET | NTRK3, ALK, ROS1, RET, NTRK1 | *EGFR* exon 19 del, *TP53* H214R |
|  |  |  | Repeat- day 57 | 4112 | 527.18 | 4100 |  | PD-L1 | CMET | NTRK3, ALK, ROS1, RET, NTRK1 | *EGFR* exon 19 del, *TP53* H214R |
| D | Ovarian | BRCA1 mutant, HRD positive | Initial- day 0 | 3315 | 454.11 | 3241 | None | PD-L1 | HER2 | N/A | BRCA1 G1706, TP53 A161T, MTOR, G1384S, TRAF7 K321N, CDKN1B Amplification, RECQL Amplification, CCND2 Amplification, DDR2 Amplification, NTRK1 Amplification, RIT1 Amplification, SDHC Amplification, PMS2 Deletion, RAC1 Deletion, RASA1 Deletion, CCND2 Amplification, DDR2 Amplification, NTRK1 Amplification, RIT1 Amplification, SDHC Amplification, PMS2 Deletion, RAC1 Deletion, RASA1 Deletion, CCNE L346R, KRAS Amplification, CD276 V330M, CSDE1 L346R, KRAS Amplification, CD276 V330M, CSDE1 K457I, DROSHA 7247M, FOXA1 E255, KMT2C K4887_F4888del, ETV6 Amplification, PIK3C2G Amplification, MED12 Deletion, ATRX Deletion, CDH1 Deletion, FTSJD1 Deletion, ZFHX3 Deletion, FLCG2 Deletion, FOXF1 Deletion, ANKRD11 Deletion, FANCA Deletion, MAP3K1 Deletion, PLK2 Deletion, CARD11 Deletion, ETV1 Deletion, NKX3-1 Deletion, DUSP4 Deletion, NSD3 Deletion, FGFR1 Deletion, BTK Deletion, PIK3C Deletion, SMAD2 Deletion, SMAD4 Deletion, MALT1 Deletion, PMAIP1 Deletion, BCL2 Deletion, SERPINB4 Deletion, SERPINB3 Deletion, SOS1 Deletion, ABRAXAS1 Deletion, EIF4E Deletion, TET2 Deletion |
| G | Breast |  | Initial- day 0 | 0 | 0 | 0 | N/A | N/A | N/A | N/A | *HER2* amplification, *TP53* R213L |
| J | Lung | - KEAP1 G509W, STK11 exon 1 loss, APC G721*, PD-L1 negative | Initial- day 0 | 4 | 0.62 | 3 | None | PD-L1 | None | ALK, POS1, CMET, RET, NTRK1, NTRK3 | None |
|  |  |  | Repeat- day 48 | 0 | 0 | 0 | N/A | N/A | N/A | N/A | None |
|  |  |  | Repeat- day 56 | 0 | 0 | 0 | N/A | N/A | N/A | N/A | None |
| K | Esophageal adenocarcinoma |  | Initial- day 0 | 84,092 | 12,252.88 | 84,092 | none | ER, PD-L1, PR | HER2 | NTRK1, NTRK3 | NGS on CSF not completed |
| L | Endometrial |  | Initial- day 0 | 4 | 0.57 | 2 | none | ER,PD-L1, PR | none | HER2, NTRK1, NTRK3, FGFR1 | NGS on CSF not completed |

Table 4: Biocept results for patients with positive CTC reports or NGS results; patients with negative CTC and NGS not listed.
